# Goethite dissolution by acidophilic bacteria

**DOI:** 10.3389/fmicb.2024.1360018

**Published:** 2024-05-22

**Authors:** Srdjan Stanković, Axel Schippers

**Affiliations:** ^1^Federal Institute for Geosciences and Natural Resources (BGR), Hannover, Germany; ^2^Faculty of Biology, University of Belgrade, Belgrade, Serbia

**Keywords:** goethite, iron(hydr)oxide, ferric iron reduction, *Acidithiobacillus*, *Acidiphilium*

## Abstract

Previous studies have reported the role of some species of acidophilic bacteria in accelerating the dissolution of goethite under aerobic and anaerobic conditions. This has relevance for environments impacted by acid mine drainage and for the potential bioleaching of limonitic laterite ores. In this study, natural well-characterized goethite mineral samples and synthetic goethite were used in aerobic and anaerobic laboratory batch culture incubation experiments with ferric iron-reducing, acidophilic bacteria, including the lithoautotrophic species *Acidithiobacillus (At.) thiooxidans*, *At. ferrooxidans,* and *At. caldus*, as well as two strains of the organoheterotrophic species *Acidiphilium cryptum*. All bacteria remained alive throughout the experiments and efficiently reduced soluble ferric iron in solution in positive control assays. However, goethite dissolution was low to negligible in all experimental assays with natural goethite, while some dissolution occurred with synthetic goethite in agreement with previous publications. The results indicate that ferric iron-reducing microbial activity at low pH is less relevant for goethite dissolution than the oxidation of elemental sulfur to sulfuric acid. Microbial ferric iron reduction enhances but does not initiate goethite dissolution in very acidic liquors.

## 1 Introduction

Goethite is one of the most frequently occurring iron(hydr)oxide minerals in the environment (Cornell and Schwertmann, 2003). Its formation and dissolution are related to biogeochemical redox processes, and the role of microorganisms in these processes has been extensively studied in environments with circumneutral pH such as soils and sediments. Published findings indicate that neutrophilic ferric iron-reducing bacteria such as *Shewanella* or *Geobacter* are able to produce significant quantities of ferrous iron in anaerobic soil and subsurface environments, where crystalline ferric iron oxides such as goethite are available for microbial reduction (Roden and Zachara, 1996; Bousserrhine et al., 1999; Roden and Urrutia, 2002; Zhang et al., 2020; Kappler et al., 2021).

Microbial ferric iron reduction has also been described for environments with low pH (Johnson et al., 1993, 2012; Küsel et al., 2002; Blöthe et al., 2008). Ferric iron reduction by acidophilic bacteria and archaea has recently been reviewed by Malik and Hedrich (2022). The first evidence that acidophilic bacteria posses the ability to reduce ferric iron under aerobic and anaerobic conditions was reported by Brock and Gustafson (1976). They showed that *Acidithiobacillus (At.) ferrooxidans* can couple sulfur oxidation with ferric iron reduction under anaerobic conditions and that the sulfur-oxidizer *At. thiooxidans* is able to reduce ferric iron during sulfur oxidation under aerobic conditions. Under anaerobic or microaerobic conditions, *At. ferrooxidans* uses ferric iron as a terminal acceptor of electrons in the respiratory chain instead of oxygen (Pronk and Johnson, 1992; Malik and Hedrich, 2022), but the mechanism of ferric iron reduction under aerobic conditions with *At. thiooxidans* is not clear. Most likely, it is a chemical reaction between ferric iron and reduced inorganic sulfur compounds such as hydrogen sulfide, generated during the bacterial oxidation of elemental sulfur to sulfuric acid (Breuker and Schippers, 2024).

Johnson and McGinness (1991) demonstrated that many strains of acidophilic heterotrophs can reduce ferric iron to some extent under aerobic conditions. Bridge and Johnson (2000) reported that the acidophilic heterotrophic bacterium *Acidiphilium cryptum* SJH can reduce ferric iron under anaerobic conditions and promote the dissolution of synthetic goethite, although the final concentrations of soluble iron in goethite dissolution experiments were very low. No dissolution of hematite was observed. Hallberg et al. (2011) and du Plessis et al. (2011) suggested that the ability of *At. ferrooxidans* to reduce ferric iron under anaerobic conditions could be exploited to enhance and accelerate the extraction of nickel and cobalt from limonitic nickel laterites in a reductive bioleaching process in which ferric iron reduction (and generation of acidity) is coupled to the oxidation of added elemental sulfur. Since the process is net-consumptive of protons, additional inputs of acidity for the critical primary reaction (acid dissolution of the minerals within which nickel and cobalt were deported) are required (Johnson and du Plessis, 2015; Johnson et al., 2021; Santos and Schippers, 2023). Laterites are supergene ore bodies that host about 70% of global nickel reserves (Stanković et al., 2020). Limonitic laterites constitute of iron oxide minerals. Goethite is usually the dominant mineral in limonites and hosts the majority of nickel. This metal is “locked” in the crystal lattice of goethite, where it substitutes iron atoms. Extensive research on bacterial dissolution of laterites in bioreactors has been conducted as reviewed by Roberto and Schippers (2022) and Santos and Schippers (2023). Stanković et al. (2022) carried out a detailed and comprehensive chemical and mineralogical characterization of original samples and bioleaching residues and showed that iron oxide phases (goethite, hematite) were almost intact after bioleaching experiments with sulfur-oxidizing bacteria under anaerobic as well as aerobic conditions. The authors concluded that the dissolution kinetics of iron oxide minerals depended on the concentration of sulfuric acid in solution.

The purpose of this study was to use natural well-characterized goethite mineral samples in addition to synthesized ones (Bridge and Johnson, 2000) and to test the ability of acidophilic bacteria to induce the dissolution of goethite under aerobic and anaerobic conditions in order to estimate the effect of a biological ferric iron reduction on the dissolution of goethite at low pH. If the dissolution of natural goethite depends on the ferric iron-reducing activity of organisms at low pH, this is very relevant for metal bioleaching of oxide ores such as limonitic laterites.

## 2 Materials and methods

### 2.1 Goethite samples

Three natural goethite mineral samples for the experiments were selected from the BGR mineral collection and analyzed as described elsewhere and briefly described below (Kaufhold et al., 2022; Stephan Kaufhold, personal communication). Goethite sample S018 originated from Conakry, Guinea; sample S337 was obtained from the Sasik mine in Pamir, Tajikistan; and sample S343 was taken from the Wolf mine in Herdorf, Germany. The samples were ground to a fine powder in a mortar and mineralogically analyzed. For all three samples, crystalline goethite (a-FeOOH) was confirmed as main mineral phase by X-ray diffraction (XRD) analysis with Rietveld refinement. Besides goethite, sample S018 contained some gibbsite (Al(OH_3_)), and sample S337 contained 1% lepidocrocite, while sample S343 consisted of pure goethite. The chemical composition was analyzed via XRF, and the results are shown in Table 1.

**Table 1:** Elemental composition of natural goethite samples (Kaufhold et al., 2022, personal communication).

Sample	SiO2, %	TiO2, %	Al2O3, %	Fe2O3, %	MnO, %	MgO, %	CaO, %	As, ppm	Ba, ppm	Bi, ppm	Ce, ppm	Co, ppm	Cr, ppm
**S343**	2	0	0	85	0	0	0	66	<57	8	<27	22	<6
**S337**	2	0	1	84	0	0	0	84	190	30	88	<9	<6
**S018**	1	0	12	70	0	0	0	32	11	16	20	28	18,012

Synthetic goethite was prepared as previously described (Atkinson et al., 1977; Lovley and Phillips, 1986; Bridge and Johnson, 2000), and the sample was stored for about 5 years (sample sG2). The synthesis was repeated just before starting the experiments to obtain a second synthetic goethite sample (sample sG1). Both samples were analyzed via XRD, infrared analysis (IR), differential thermal analysis (DTA), and the BET gas adsorption technique according to previous studies (Houben and Kaufhold, 2011; Kaufhold et al., 2022). All methods detected goethite as the main mineral phase. The BET data (Table 2) showed a much higher specific surface area for the synthetic than for the natural goethite samples, which indicates a lower crystallinity of the synthetic goethite and the presence of ferrihydrite, while the natural goethite samples were well crystalline (Kaufhold et al., 2022).

**Table 2:** BET data of the specific surface area (m^2^ g^−1^) of the goethite samples.

Natural goethite		Synthetic goethite	
nG S337	nG S018	sG1	sG2
19	35	62	97

### 2.2 Acidophilic bacteria

Acidophilic, chemolithoautotrophic, and chemoorganoheterotrophic bacteria were taken from the BGR culture collection. The autotrophic species *Acidithiobacillus* (*At.*) *thiooxidans* DSM 14887^T^, *At. ferrooxidans* DSM 14882^T^, and *At. caldus* DSM 8584^T^ were cultivated in basal salts medium at pH 2 supplemented with trace elements (HBS-TE, Wakeman et al., 2008) and 1% elemental sulfur or 25 mM ferrous sulfate in case of *At. ferrooxidans*. All organisms were grown in sterile medium aerobically on a shaker at 30°C besides *At. caldus* at 45°C.

Two strains of the heterotrophic species *Acidiphilium (A.) cryptum* (the type strain DSM 2389^T^ and strain SJH; Bridge and Johnson, 2000) were grown under aerobic conditions in a medium with the following concentrations (g/L) of components: (NH_4_)_2_SO_4_, 0.15; MgSO_4_, 0.50; KCl, 0.05; KH_2_PO4, 0.05; Ca(NO_3_)_2_, 0.01, and yeast extract, 0.2. Glycerol (10 mM) was added as carbon and energy source; 25 mM soluble ferric sulfate was added; and the medium was adjusted to pH 2.0 with 25% (v/v) sulfuric acid. The bacteria were incubated in sterile medium at 30°C, not shaken (Bridge and Johnson, 2000).

### 2.3 Goethite dissolution experiments

Three series of experiments were carried out. Only a small amount of goethite samples was obtained from the BGR mineral collection. Sample S343 was used up for series 1 experiments; thus, two other goethite samples were used for further experiments.

Series 1: *At. thiooxidans and At. caldus* with goethite sample S343 each, aerobic.

Series 2: *At. ferrooxidans* with goethite samples S018 and S337 each, anaerobic.

Series 3: *A.cryptum* with goethite samples S018 and S337 each, anaerobic.

Series 4: *A.cryptum* with goethite sample S337, and the two synthetic goethite samples each, anaerobic.

Regularly, liquid samples were taken from all experimental assays and analyzed with electrodes for pH and redox potential vs. Ag/AgCl reference in a glass (combination) electrode. Ferrous and ferric iron concentrations were measured in 0.2 μm-filtered samples using the Ferrozine assay (Lovley and Phillips, 1986). Cells were counted using a counting chamber under a phase-contrast microscope at 630x magnification.

Series 1: *At. thiooxidans* and *At. caldus* were first cultivated in HBS-TE medium at initial pH 3 supplied with 1 g/L of elemental sulfur (S^0^) each. After 10 days of cultivation, the bacterial cultures were used as inoculum (10% v/v). Three flasks containing 0.5% w/v of fine-grained S343 goethite sample and 1 g/L of S^0^ were inoculated with an *At. thiooxidans* culture and put on an orbital shaker with 120 rpm at 30°C. Three shake flasks with goethite sample were inoculated with *At. caldus* and cultivated at a temperature of 45°C. Negative controls were abiotic assays without cell inoculation in duplicates at 30°C and 45°C. The duration of the experiment was five weeks.

Series 2: *At. ferrooxidans* was first cultivated under anaerobic conditions in HBS/TE pH 2 with 25 mM ferric sulfate and 1 g/L S^0^ in gas-tight flasks with a N_2_/CO_2_ (80%/20%, v/v) gas mixture at 30°C (not shaken). After 18 days, the ferric iron-reducing cells served as inoculum (10% v/v) for the goethite dissolution experimental assays in anaerobic, gas-tight flasks. These contained as the pre-culture HBS/TE at pH 2 and 1 g/L S^0^ with a N_2_/CO_2_ gas mixture at 30°C (not shaken). Either 0.1 g (0.5% w/v) fine-grained goethite (S018 or S337) or 25 mM ferric sulfate as positive control was added as well as *At. ferrooxidans* cells. Negative controls were abiotic assays without cell inoculation. All assays run as triplicates.

At the end of the experiment after 44 days, fresh HBS medium pH 2.0 with 25 mM ferrous sulfate was inoculated from each assay and aerobically cultivated on a shaker as well as under anaerobic conditions with sulfur and ferric iron as described above to test for viability of *At. ferrooxidans*. For all aerobic biotic assays, the ferrous iron was completely oxidized to ferric iron within one week, while the ferrous iron concentration was almost not changed in the abiotic assays. Under anaerobic conditions, the ferric iron was almost completely reduced to ferrous iron in all biotic assay, while no ferrous iron was detected in the abiotic assays. This means that cells were viable in all biotic assay, while the abiotic assay remained sterile throughout the experiment.

Series 3: The experiments were designed according to Bridge and Johnson (2000). The two *Acidiphilium cryptum* strains were cultivated with the medium as described above under aerobic conditions in flasks at 30°C (not shaken). After five days, the ferric iron-reducing cells were concentrated via centrifugation, washed twice with medium, then re-suspended in medium in an anaerobic chamber, and used as inoculum (10% v/v) for the goethite dissolution experimental assays in anaerobic flasks. These contained as the pre-culture medium with glycerol at 30°C (not shaken). Either 0.1 g (0.5% w/v) fine-grained goethite (S018 or S337) or 25 mM ferric sulfate as positive control was added, as well as cells. Negative controls were abiotic assays without cell inoculation. All assays run as triplicates.

At the end of the experiment after 42 days, fresh medium was inoculated from each biotic assay and aerobically cultivated on a shaker for nine days. Cell growth was then inspected via phase contrast light microscopy, and cells were always visible which means that cells were viable in all biotic assay throughout the experiment.

Series 4: The experiments were conducted as described for series 3 but with the two synthetic goethite samples and only one natural goethite S337. This experiment was conducted for 33 days. To confirm the results, the experiment was repeated and conducted for 13 days.

## 3 Results

### 3.1 Goethite dissolution with sulfur-oxidizing acidophiles (*Acidithiobacillus* spp.) under aerobic conditions (series 1)

**Figure 1:**
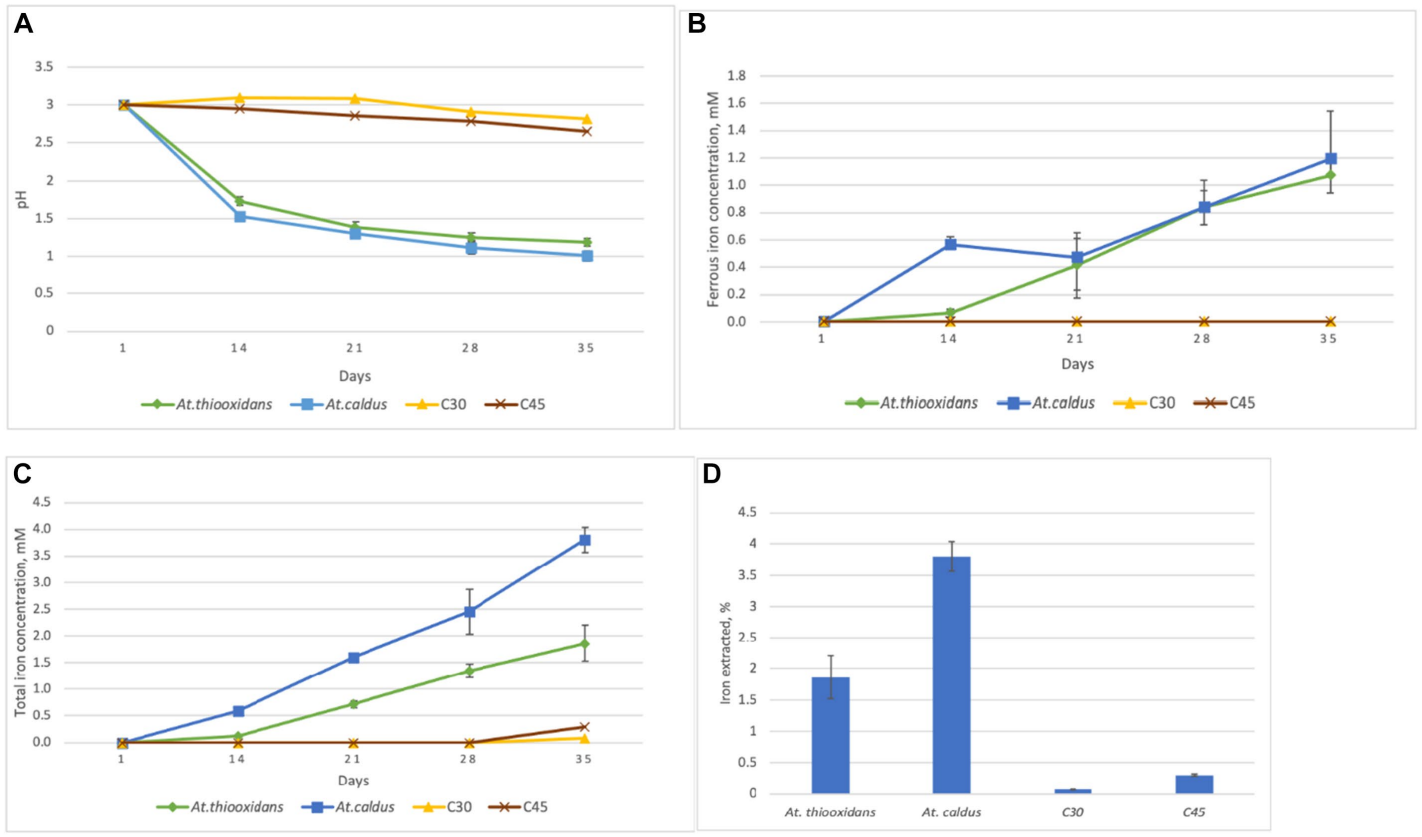
Changes of pH **(A)** ferrous iron concentration **(B)** total iron concentration **(C)** and total iron extraction **(D)** for the aerobic goethite S343 dissolution experiment with *At. thiooxidans* at 30°C and *At. caldus* at 45°C as well as for the not inoculated, abiotic control assays at the same temperature, respectively, C30 and C45. Mean values and standard deviation for three parallel assays are shown each.

Changes of pH, ferrous iron, and total iron concentrations during the experiment are presented in Figure 1. The pH strongly decreased in the biotic assays, while only a slight decrease was observed for the not inoculated, abiotic control assays. The final pH value in flasks inoculated with *At. thiooxidans* was 1.2, and in flask inoculated with *At. caldus,* it was 1.0. Ferrous iron and total iron concentrations steadily increased for the biotic assays but remained very low for the abiotic assays. At the end of the experiment, the mean total iron concentrations in flasks inoculated with *At. thiooxidans* and *At. caldus* were 1.9 mM and 3.8 mM, respectively, and total iron concentrations in control flasks at 30°C and 45°C were 0.08 mM and 0.3 mM, respectively. The mean ferrous iron fraction of the total iron concentration at the end of the experiment was 54 ± 4.45% in flasks inoculated with *At. thiooxidans* and 34 ± 2.25% in flasks inoculated with *At. caldus*.

**Figure 2:**
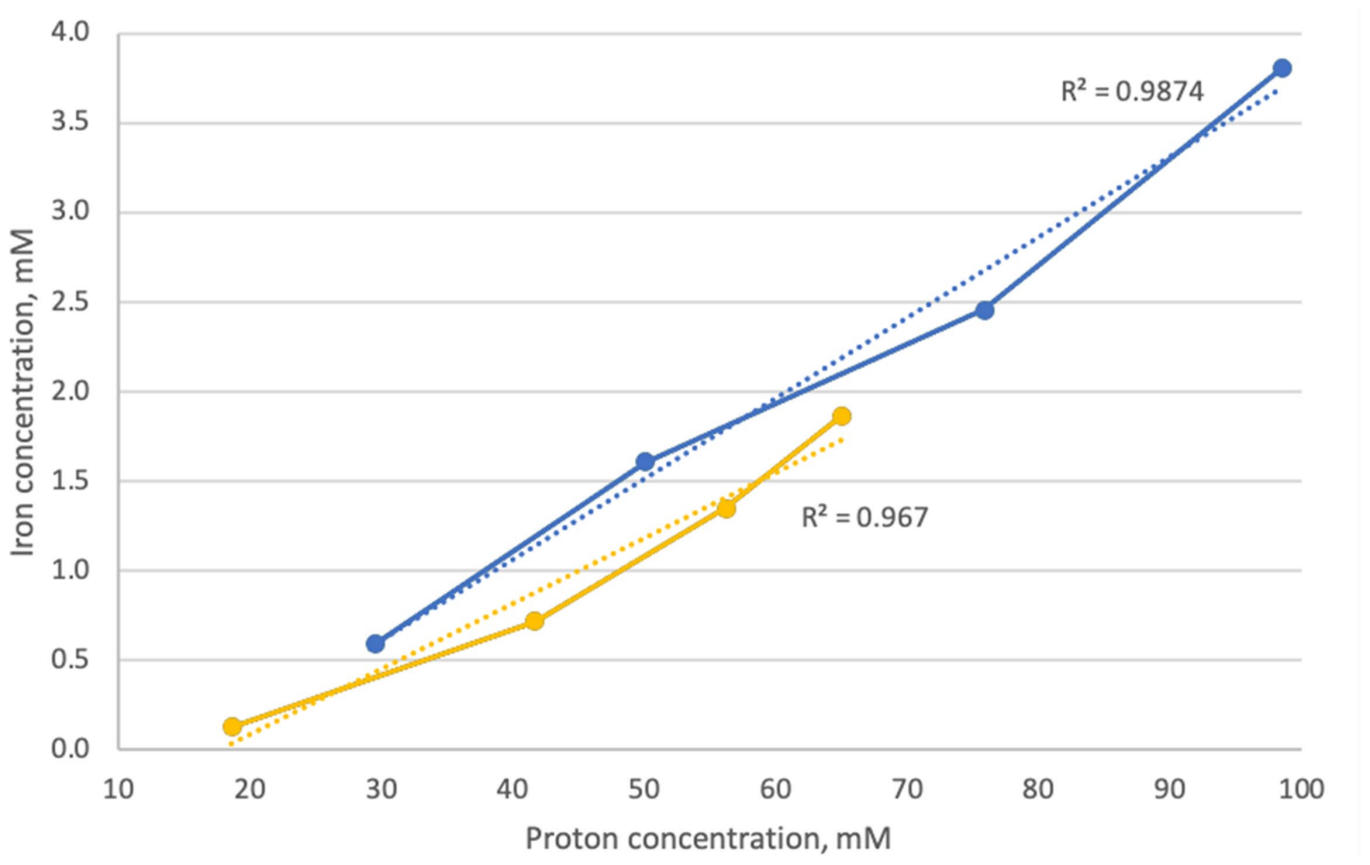
Correlation between extracted iron and proton concentration during goethite dissolution experiments in inoculated flasks with *At. caldus* (blue) and *At. thiooxidans* (yellow).

Figure 2 shows the relationship and correlation coefficients between total iron extraction and concentration of protons in the solution, calculated from pH values for the biotic assays.

### 3.2 Goethite dissolution with *At. ferrooxidans* under anaerobic conditions (series 2)

**Figure 3:**
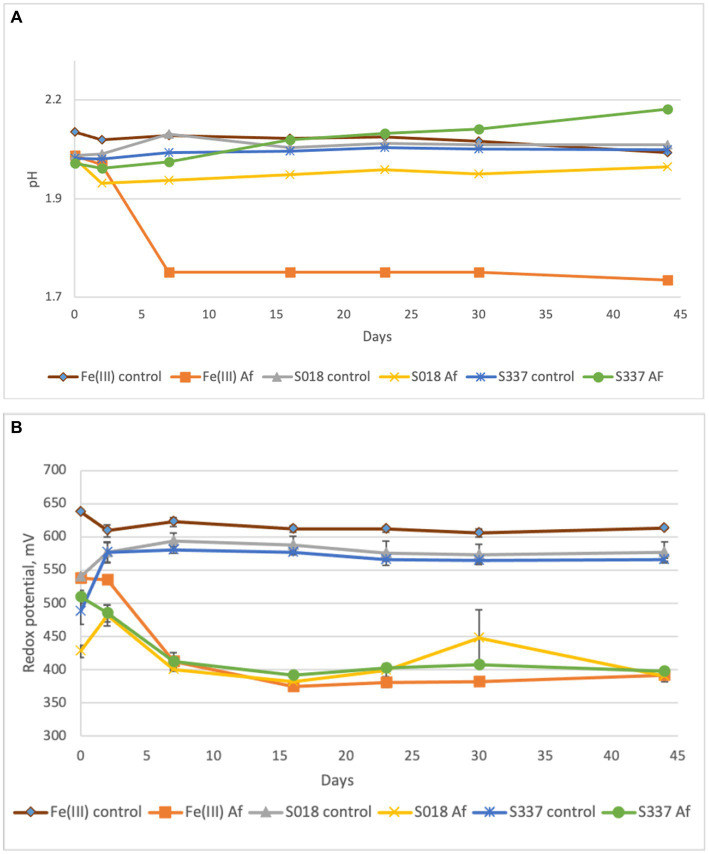
Changes of pH **(A)** and redox potential vs. Ag/AgCl **(B)** of the solutions during the anaerobic goethite dissolution experiment with *At. ferrooxidans*. Fe(III) control—not inoculated flasks with 25 mM ferric iron, Fe(III) Af—inoculated positive control with 25 mM ferric iron, S018 control—not inoculated control with S018 goethite sample, S018 Af—inoculated flasks with S018 goethite sample, S337 control—not inoculated control with S337 goethite sample, S337 Af—inoculated flasks with S337 goethite sample. Presented data are mean values of three independent experiments with standard deviations.

After seven days of experimental time, the pH of the inoculated positive control flasks with 25 mM ferric iron (Fe(III) Af) dropped to 1.7, indicating microbial activity. The pH of the solution in inoculated flask with the S018 goethite sample slightly decreased (S018 Af), and the pH in flasks with the S337 sample (S337 AF) slightly increased (Figure 3A). As a result of ferric iron reduction, the redox potential decreased in all inoculated assays in comparison with uninoculated controls (Figure 3B).

**Figure 4:**
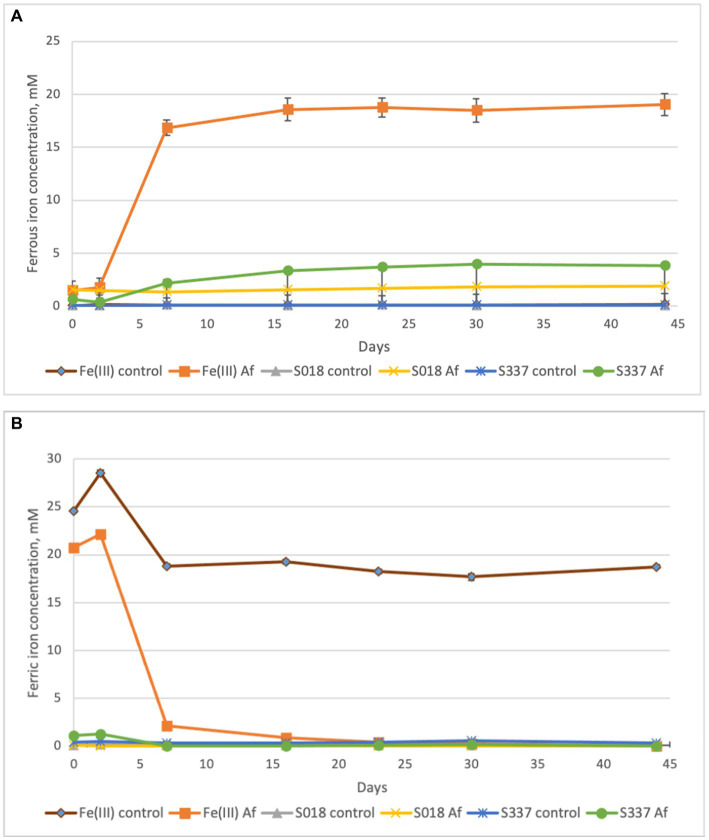
Changes in concentrations of ferrous **(A)** and ferric iron **(B)** in the solutions during the anaerobic goethite dissolution experiment with *At. ferrooxidans*. Fe(III) control—not inoculated flask with 25 mM ferric iron, Fe(III) Af—inoculated positive control with 25 mM ferric iron, S018 control—not inoculated control with S018 goethite sample, S018 Af—inoculated flask with S018 goethite sample, S337 control—not inoculated control with S337 goethite sample, S337 Af—inoculated flask with S337 goethite sample.

**Figure 5:**
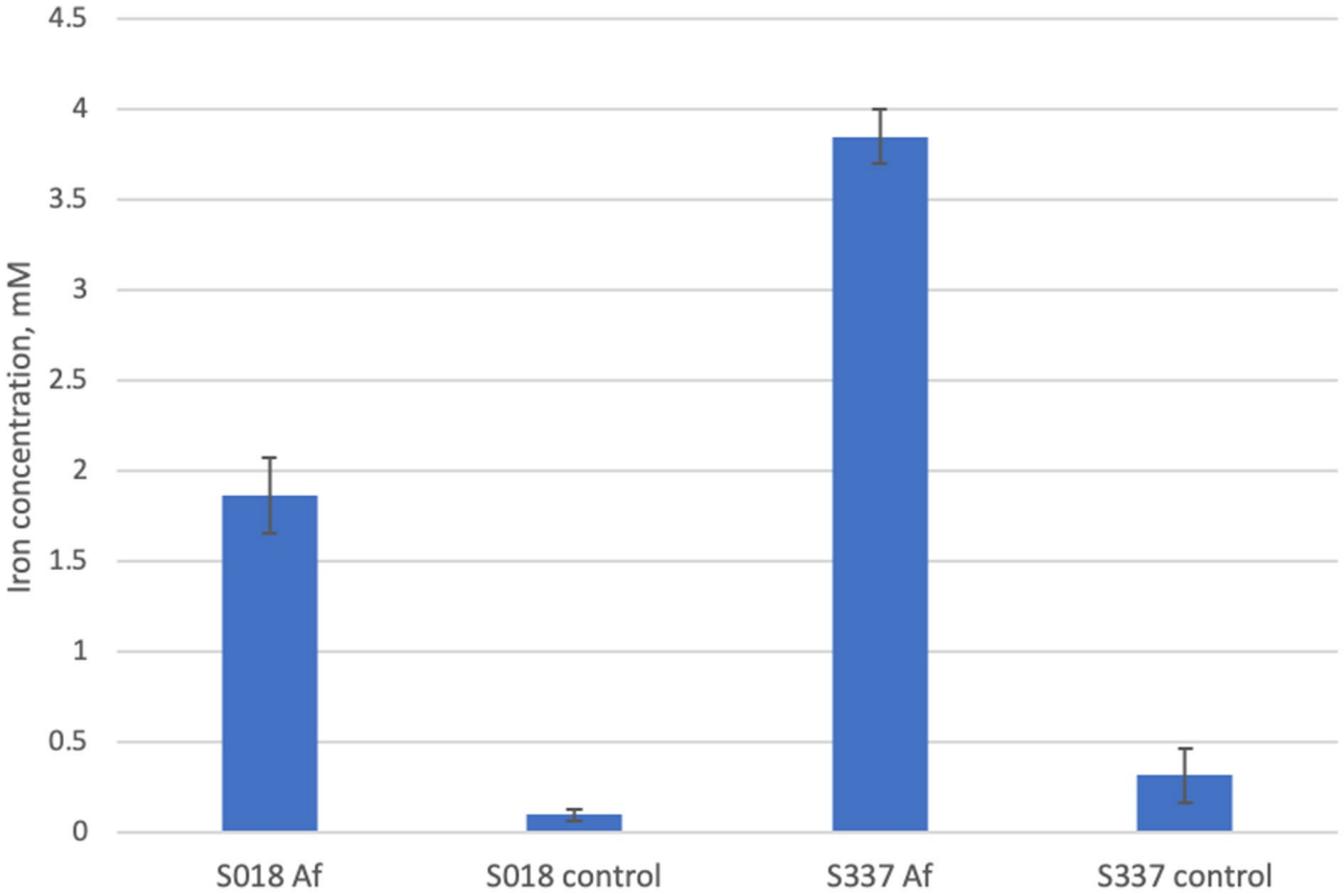
Total iron extraction after the anaerobic goethite dissolution experiment with *At. ferrooxidans* and the two goethite samples S018 and S337.

In the inoculated positive control assays with 25 mM ferric iron, all ferric iron was reduced to ferrous iron, confirming ferric iron-reducing microbial activity. In inoculated flasks with goethite, some iron extraction occurred, and all extracted iron was reduced to ferrous iron (Figure 4). In comparison with the uninoculated control assays, iron extraction in inoculated samples was somehow higher (Figure 5).

### 3.3 Goethite dissolution with heterotrophic acidophiles (*Acidiphilium cryptum*) (series 3)

**Figure 6:**
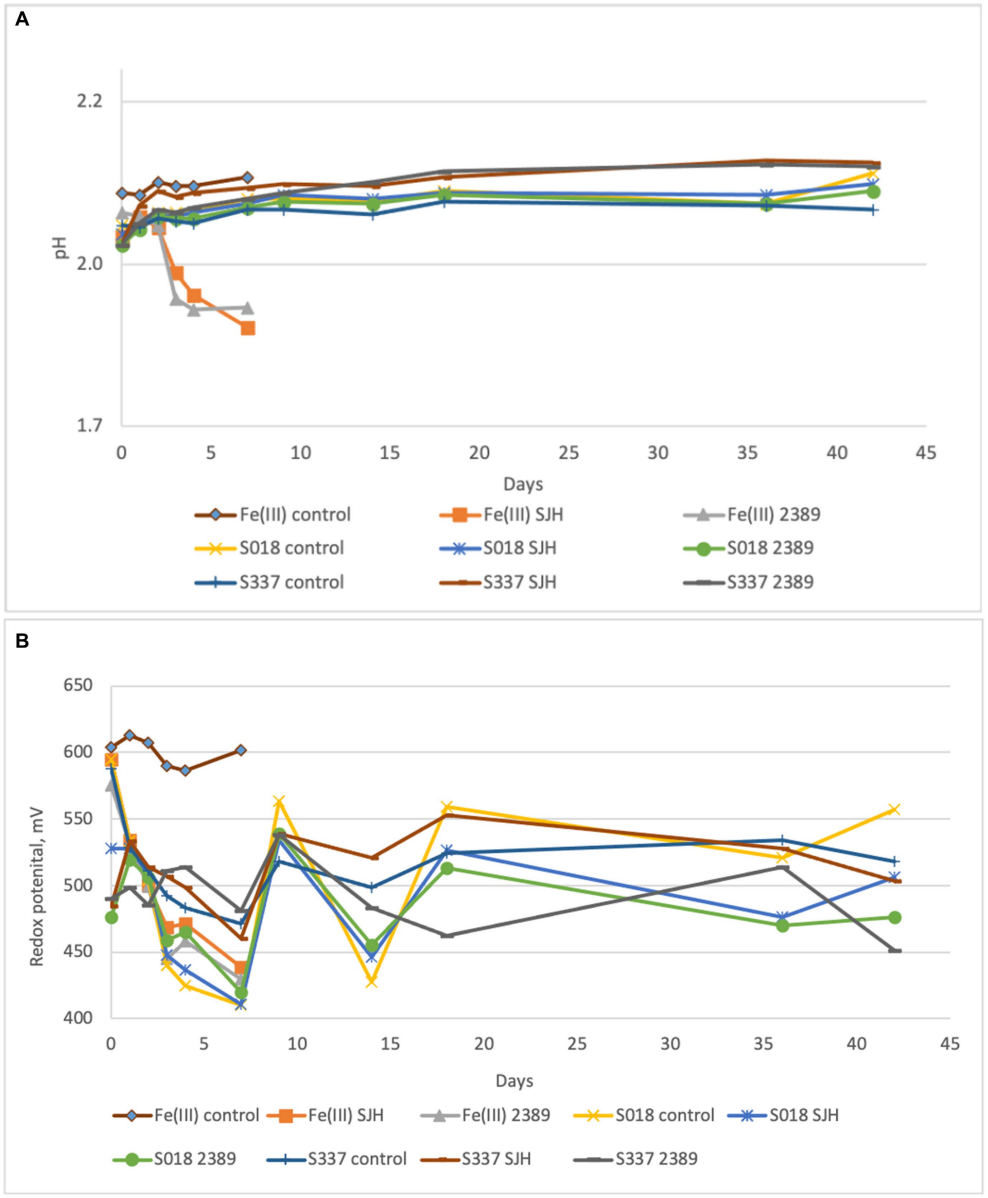
Changes in pH **(A)** and redox potential vs. Ag/AgCl **(B)** of the solutions during the anaerobic goethite dissolution experiment with the *Acidiphilium cryptum* strains SJH and DSM 2389^T^ and the two goethite samples S018 and S337. Fe(III) control—not inoculated control with 25 mM ferric iron, Fe(III) SJH inoculated control with *A. cryptum* SJH and 25 mM ferric iron, Fe(III) 2389 inoculated control with *A. cryptum* DSM 2389^T^ and 25 mM ferric iron, S018 control—not inoculated control with S018 goethite sample, S018 SJH—inoculated flask with *A. cryptum* SJH and S018 sample, S018 2389 inoculated flask with *A. cryptum* DSM 2389^T^ and S018 sample, S337 control—not inoculated control with S337 goethite sample, S337 SJH—inoculated flask with *A. cryptum* SJH and S337 sample, S337 2389— inoculated flask with *A. cryptum* DSM 2389^T^ and S337 sample.

**Figure 7:**
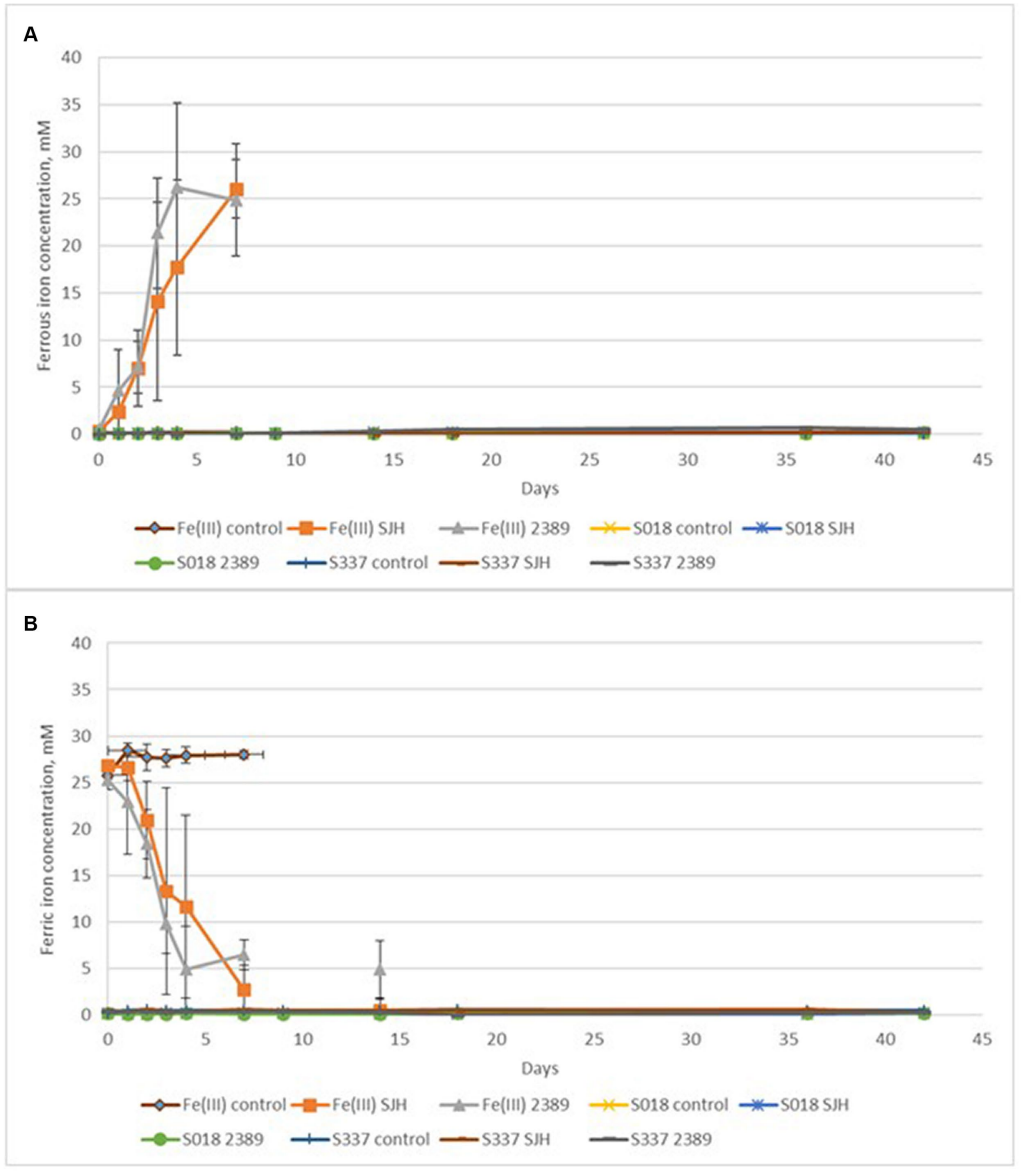
Changes in ferrous **(A)** and ferric **(B)** iron concentrations during the anaerobic goethite dissolution experiment with the *Acidiphilium cryptum* strains SJH and DSM 2389^T^ and the two goethite samples S018 and S337. Fe(III) control—not inoculated control with 25 mM ferric iron, Fe(III) SJH inoculated control with *A. cryptum* SJH and 25 mM ferric iron, Fe(III) 2389 inoculated control with *A. cryptum* DSM 2389^T^ and 25 mM ferric iron, S018 control—not inoculated control with S018 goethite sample, S018 SJH—inoculated flask with *A. cryptum* SJH and S018 sample, S018 2389 inoculated flask with *A. cryptum* DSM 2389^T^ and S018 sample, S337 control—not inoculated control with S337 goethite sample, S337 SJH—inoculated flask with *Acidiphilium* SJH and S337 sample, S337 2389—inoculated flask with *A. cryptum* DSM 2389^T^ and S337 sample.

In the inoculated positive control flasks with 25 mM ferric iron, both *Acidiphilium cryptum* strains (SJH and DSM 2389^T^) were able to reduce ferric to ferrous iron and to decrease the pH of the solution (Figure 6A) confirming ferric iron-reducing microbial activity. The redox potential only remained high in the chemical Fe(III) control assays (Figure 6B). Overall, the amount of extracted iron from inoculated flasks with the natural goethite samples was negligible (Figure 7), and there was no significant difference in iron extraction between inoculated and uninoculated flasks with goethite samples.

### 3.4 Goethite dissolution with heterotrophic acidophiles (*Acidiphilium cryptum*) including synthetic goethite (series 4)

**Figure 8:**
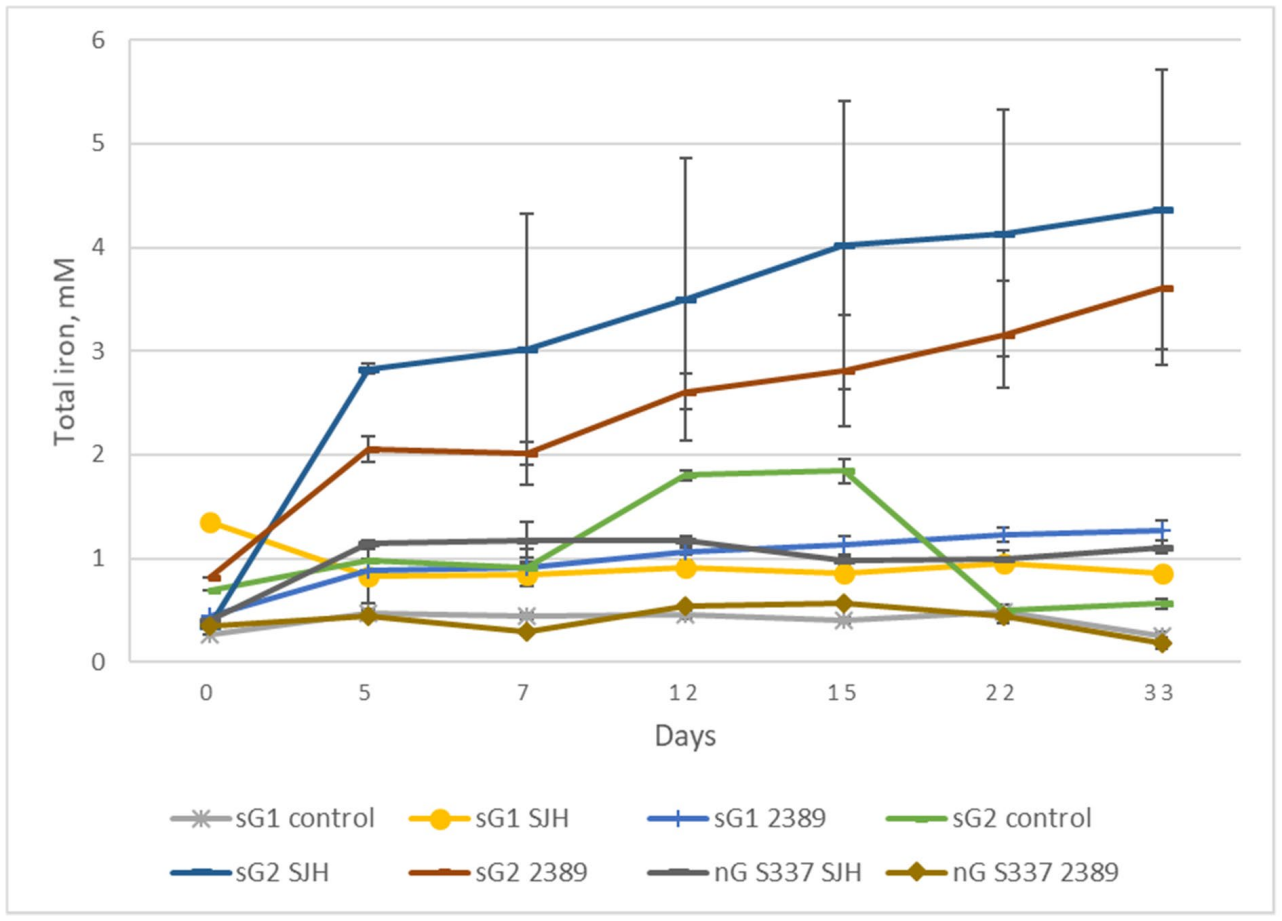
Changes in total iron concentrations during the second anaerobic goethite dissolution experiment with the *Acidiphilium cryptum* strains SJH and DSM 2389^T^, the natural goethite (nG) sample S337, and two synthetic goethite samples (sG1 and sG2).

**Figure 9:**
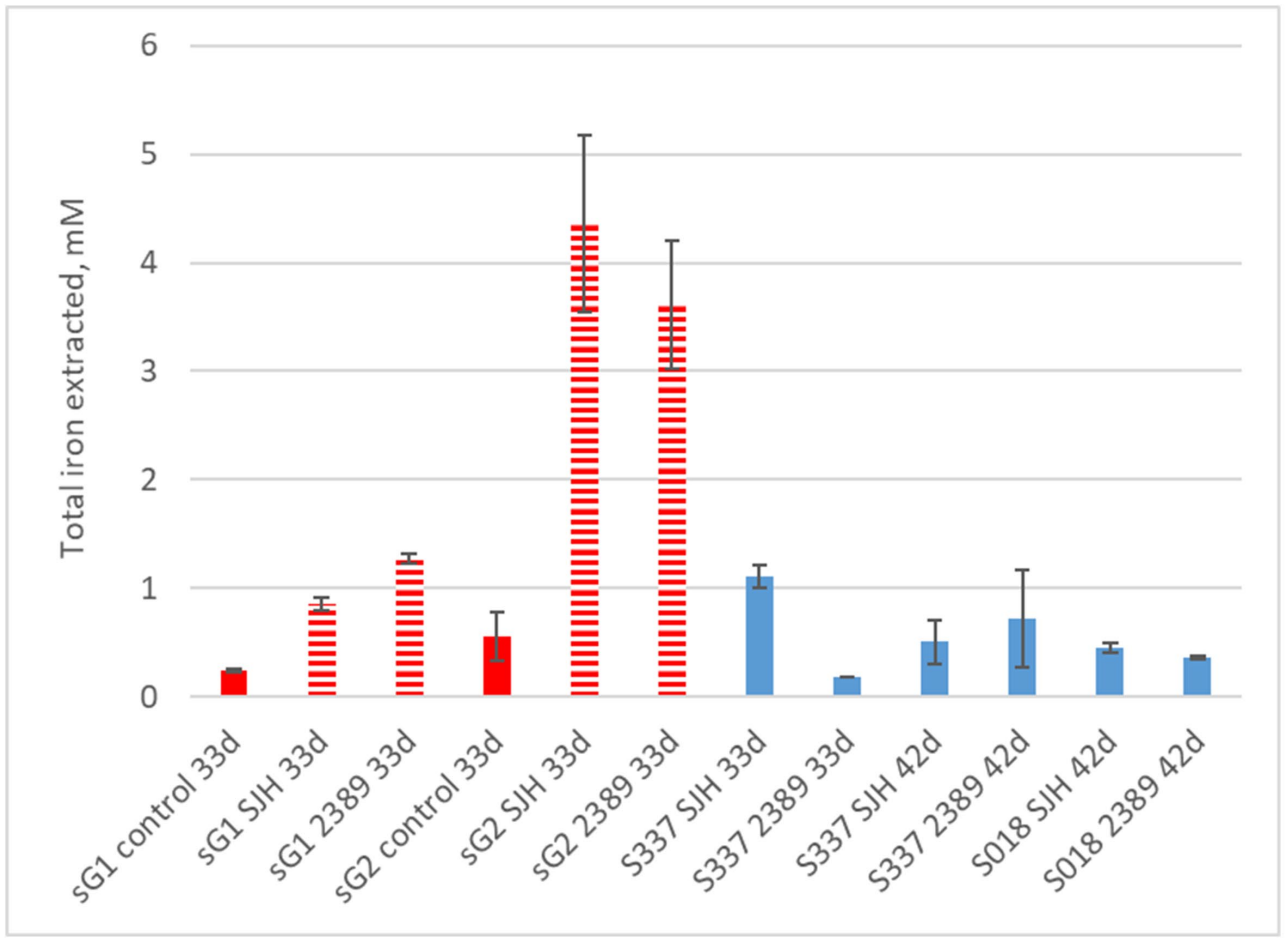
Total iron extraction after two anaerobic goethite dissolution experiments running for 33 days and 42 days, respectively, with the *Acidiphilium cryptum* strains SJH and DSM 2389 ^T^ and the natural goethite (nG) samples S018 and S337 (blue) and two synthetic goethite samples (sG1 and sG2) (red).

In contrast to the natural goethite (S337), for which mineral dissolution was not detectable, iron dissolution was detectable over 33 days for one of the two synthetic goethite samples (Figure 8). A comparison of total iron extraction after the two anaerobic goethite dissolution experiments running for 33 days and 42 days, respectively, with two natural and synthetic goethite samples each are shown in Figure 9.

## 4 Discussion

In this study, natural well-characterized goethite mineral samples and synthetic goethite were used in aerobic and anaerobic laboratory batch culture incubation experiments with ferric iron-reducing, acidophilic bacteria, including the lithoautotrophic species *Acidithiobacillus thiooxidans, At. ferrooxidans,* and *At. caldus*, as well as the organoheterotrophic species *Acidiphilium cryptum*. All bacteria remained alive throughout the experiments and efficiently reduced soluble ferric iron in solution in positive control assays. While the sulfur-oxidizing *Acidithiobacillus* species showed some dissolution of natural goethite under anaerobic as well as aerobic conditions reflected by higher soluble iron concentrations than in the abiotic control assays, there was no detectable dissolution of natural goethite with the organoheterotrophic *A. cryptum*. In contrast, in *A. cryptum* assays with synthetic goethite, ferric iron reduction was measurable in agreement with previous findings (Bridge and Johnson, 1998, 2000). However, synthetic goethite contains the much easier dissolvable ferrihydrite as the precursor mineral in the goethite formation process being hardly detectable via XRD. The presence of ferrihydrite in the synthetic goethite samples was revealed by the higher specific surface area than that for natural goethite (Table 2; Houben and Kaufhold, 2011; Kaufhold et al., 2022). Thus, there is limited relevance of the experiments with synthetic goethite for exploring the dissolution behavior of natural goethite. Goethite dissolution by acidophilic bacteria might be based on three processes (Johnson et al., 2021; Vera et al., 2022):

1. Microbial acid generation via sulfur or organic carbon oxidation, leading to protonation of goethite (Pyzik and Sommer, 1981; Wieland et al., 1988; Schwertmann, 1991; Cornell and Schwertmann, 2003; McDonald and Whittington, 2008; Senanayake et al., 2011; Klyukin et al., 2018)
2. Microbial ferric iron reduction coupled to sulfur or organic carbon oxidation, shifting the equilibrium between goethite solid-phase and soluble ferric iron (Bridge and Johnson, 2000; Hallberg et al., 2011) with likely less relevance due to the very low solubility product of goethite (Schwertmann, 1991), which requires protonation for breaking Fe − O bonds for mineral dissolution (Klyukin et al., 2018)

In the case of acidophilic sulfur-oxidizers (*Acidithiobacillus*) as well as acidophilic organoheterotrophs (*Acidiphilium*), both processes occur simultaneously and can be described by these equations (Pronk et al., 1991; Schwertmann, 1991; Coupland and Johnson, 2008):


(1) $$\mathrm{FeOOH}+3\;H^{+}\to {Fe}^{3+}+2\;H_{2}O$$

(2) $$S^{0}+6\;{Fe}^{3+}+4\;H_{2}O\to {{\mathrm{SO}}_{4}}^{2-}+6\;{Fe}^{2+}+8\;H^{+}$$

(3) $$C_{6}H_{12}O_{6}+4\;{Fe}^{3+}+6\;H_{2}O\to 24\;{Fe}^{2+}+6\;{CO}_{2}+24\;H^{+}$$

Reactions 2, 3 are dependent on Reaction 1, i.e., acid dissolution of goethite, to generate soluble ferric iron and do not work in isolation. Considering that the goethite solubility is a function of pH and several orders of magnitude higher at pH 2 than at pH 7 (Cornell and Schwertmann, 2003), the relevance of microbial ferric iron reduction for goethite dissolution is much higher for neutrophilic bacteria than for acidophiles, meaning that the ferric iron-reducing activity of acidophiles is likely superimposed by protonation of goethite. Microbial ferric iron reduction enhances but does not initiate goethite dissolution in very acidic liquors.

There is a third mechanism based on chemical reduction of goethite (Pyzik and Sommer, 1981; Schwertmann, 1991; Klyukin et al., 2018):


(4) $$\mathrm{FeOOH}+e^{-}+3\;H^{+}\to {Fe}^{2+}+2\;H_{2}O$$

A chemical reduction of the solid phase ferric iron might occur during microbial sulfur oxidation by releasing reducing inorganic sulfur compounds such as hydrogen sulfide (Osorio et al., 2013; Breuker and Schippers, 2024), delivering the electrons by their oxidation according to the following equation as one possibility (Pyzik and Sommer, 1981; Morse et al., 1987):


(5) $$H_{2}S+2\;\mathrm{FeOOH}+4\;H^{+}\to S^{0}+4\;H_{2}O+2\;{Fe}^{2+}$$

However, according to Eqs. 4, 5, the reduction goes along with proton consumption (acid dissolution) as well; thus, all three mechanisms occur simultaneously, and reducing inorganic sulfur compounds would also chemically reduce soluble ferric iron to ferrous iron, making it impossible to distinguish this process from dissimilatory ferric iron reduction by just measuring changes of ferric or ferrous iron concentrations over time. Based on this overall considerations, the particular results of our study are discussed in the following.

During the aerobic natural goethite dissolution experiment with sulfur-oxidizing acidophiles *At. thiooxidans* and *At. caldus* (series 1), the pH of the solution with goethite samples dropped to approximately 1 due to the biogenic production of sulfuric acid, and substantial amounts of ferrous iron were produced. In order to determine whether goethite dissolution (i.e., iron extraction) was a function of the acid concentration, correlation coefficients between iron extraction and concentration of protons (H^+^ ions) were calculated. High correlation coefficients between total iron extraction and proton concentration in flasks inoculated with *At. thiooxidans* (*r*^2^ = 0.93) and *At. caldus* (*r*^2^ = 0.97) strongly indicate that dissolution of goethite was mainly a result of acid dissolution. However, during oxidation of elemental sulfur by these acidophilic sulfur-oxidizers, hydrogen sulfide was detected as a sulfur compound intermediate (Breuker and Schippers, 2024). Such inorganic sulfur compounds would also reductively reduce iron(hydr)oxides (Schippers and Jørgensen, 2001; Johnson et al., 2021) not just reduce soluble ferric iron to ferrous iron. This means that the sulfur oxidation by *Acidithiobacillus* species provides both protons and reduced inorganic sulfur compounds, and both are contributing to goethite dissolution.

During the anaerobic natural goethite dissolution experiment with *At. ferrooxidans,* the amount of total iron extracted was significantly higher in inoculated flasks in comparison with not inoculated, abiotic controls. Oxidation of elemental sulfur under anaerobic conditions also produces hydrogen sulfide (Osorio et al., 2013) and sulfuric acid. The pH in the positive control inoculated flasks with 25 mM ferric iron dropped to 1.7, but in inoculated flasks with goethite samples, the pH remained around a value of 2 during the experiment. This missing shift of pH can be explained by a relatively low efficiency of sulfur oxidation by *At. ferrooxidans* under anaerobic conditions in comparison with aerobic sulfur oxidation by *At. thiooxidans* and *At. caldus*—at pH 2 aerobic sulfur oxidation provides approximately 3.6 times more free energy in comparison with anaerobic sulfur oxidation and the population of aerobic sulfur oxidizers grows more rapidly in comparison with anaerobic growth of *At. ferrooxidans* on elemental sulfur (Brock and Gustafson, 1976).

The relatively low efficiency of sulfur oxidation by *At. ferrooxidans* combined with proton consumption by goethite dissolution was the most probable explanation for a pH buffering effect. Production of sulfuric acid by *At. ferrooxidans* caused goethite dissolution, but shifting the equilibrium between goethite solid-phase and soluble ferric iron due to dissimilatory ferric iron reduction might play only a minor role because of the dominance of goethite protonation for dissolution of the mineral at low pH (see above).

Experiments with the organoheterotrophic *Acidiphilium* species are most suitable for evaluation of a putative microbial reductive dissolution of goethite, since there is no interference of the strong sulfuric acid or hydrogen sulfide produced by bacterial sulfur oxidation. The slight drop in pH values of inoculated positive controls with 25 mM ferric iron is a consequence of production of protons during anaerobic oxidation of organic molecules, where ferric iron serves as an electron acceptor (Chemical Equation 2): in inoculated flasks with natural goethite, the pH remained around a value of 2 during the experiment due to the limited amount of electron acceptors (ferric iron) available for oxidation of organic electron donors, and consumption of protons in reaction with goethite (Chemical Equation 1). As mentioned above, there was no enhanced natural goethite dissolution by *A. cryptum;* thus, ferric iron activity alone does not enhance goethite dissolution.

Our additional experiments with synthetic goethite confirmed previous data (Bridge and Johnson, 2000). These authors demonstrated in similar experiments with *Acidiphilium cryptum* SJH the dissolution of a wide range of synthetic ferric iron–containing minerals such as akaganeite, jarosite, natrojarosite, and goethite as well as amorphous Fe(OH)_3_ (ferrihydrite), while hematite was not dissolved. The discrepancy between natural and synthetic goethite in our experiments could be explained by the different specific surface area of the mineral samples (Table 2) reflecting a higher crystallinity of the natural goethite vs. the synthetic goethite likely containing ferrihydrite. However, also metal substitution (Mn, Co, Cr, Al) for Fe in goethite affects the dissolution rate (Bousserrhine et al., 1999; Houben and Kaufhold, 2011; Kaufhold et al., 2022).

## 5 Conclusion

Natural goethite dissolution was low to negligible in all experimental assays, which argues for the minor role of acidophiles in this process in agreement with a recent study on laterite bioleaching including a thorough quantitative mineralogical analysis (Stanković et al., 2022). The results indicate that ferric iron-reducing microbial activity at low pH is not directly related to the goethite dissolution rate, which is rather a function of pH. Thus, oxidation of elemental sulfur to sulfuric acid by acidophiles is more relevant for goethite dissolution than dissimilatory ferric iron reduction (despite this is coupled to inorganic sulfur compound oxidation in case of anaerobic growth of *At. ferrooxidans*). Goethite dissolution by acidophilic bacteria that used elemental sulfur as an electron donor under aerobic (*At. thiooxidans* and *At. caldus*) and anaerobic conditions (*At. ferrooxidans*) is most likely a consequence of bacterial production of sulfuric acid and sulfur compound intermediates such as hydrogen sulfide probably serving as chemical reductant for goethite. In this study, there was no effect of ferric iron reduction by the organoheterotrophic *Acidiphilium cryptum* strains on natural goethite dissolution. The contribution of bacterial ferric iron reduction to dissolution of natural goethite at low pH was negligible because a potential goethite dissolution by shifting the equilibrium between goethite solid-phase and soluble ferric iron as a result of the ferric iron-reducing activity of acidophiles is likely superimposed by a more effective goethite dissolution due to protonation of the mineral at low pH. This finding negatively affects bioleaching of limonitic laterites, which contain goethite as the main mineral phase, incorporating a large proportion of the nickel in the ore. However, acid generation and release of inorganic sulfur compounds during sulfur oxidation by acidophiles are more relevant for goethite dissolution, and thus, bioleaching of limonitic laterites remains a viable process option.

## Data availability statement

The original contributions presented in the study are included in the article/supplementary material, further inquiries can be directed to the corresponding author.

## Author contributions

SS: Conceptualization, Data curation, Formal analysis, Investigation, Methodology, Validation, Visualization, Writing – original draft, Writing – review & editing. AS: Conceptualization, Data curation, Formal analysis, Investigation, Methodology, Resources, Validation, Visualization, Writing – original draft, Writing – review & editing.
